# LB13. The Efficacy and Impact in Heathy Infants of Nirsevimab on Medically Attended RSV Lower Respiratory Tract Infection

**DOI:** 10.1093/ofid/ofab466.1649

**Published:** 2021-12-04

**Authors:** Laura Hammitt, Laura Hammitt, Ron Dagan, Yuan Yuan, Manuel Baca Cots, Miroslava Bosheva, Shabhir A Mahdi, William J Muller, William J Muller, Heather J Zar, Dennis Brooks, Amy Grenham, Ulrika Wählby Hamrén, Vaishali S Mankad, Pin Ren, Therese Takas, Jon Heinrichs, Amanda Leach, M Pamela Griffin, Tonya L Villafana

**Affiliations:** 1 Johns Hopkins School of Public Health, Baltimore, MD; 2 Ben-Gurion University of the Negev, Beer Sheva, HaDarom, Israel; 3 AstraZeneca, Gaithersburg, MD, USA, Gaithersburg, Maryland; 4 Quirónsalud Málaga Hospital, Málaga, Spain, Málaga, Andalucia, Spain; 5 University Multiprofile Hospital for Active Treatment Sv. Georgi Medical University, Plovdiv, Bulgaria, Plovdiv, Plovdiv, Bulgaria; 6 University of the Witwatersrand, Johannesburg, South Africa, Johannesburg, Gauteng, South Africa; 7 Northwestern University, Chicago, IL; 8 Red Cross Children’s Hospital and SA-MRC Unit on Child & Adolescent Health, University of Cape Town, South Africa, Cape Town, Western Cape, South Africa; 9 AstraZeneca, Gothenburg, Sweden, Mölndal, Vastra Gotaland, Sweden; 10 AstraZeneca, Gaithersburg, Maryland; 11 Sanofi Pasteur, Swiftwater, PA, USA, Swiftwater, Pennsylvania

## Abstract

**Background:**

Respiratory syncytial virus (RSV) is the most common cause of lower respiratory tract infection (LRTI) in infants. Nirsevimab is a single-dose monoclonal antibody with extended half-life that was shown to protect preterm infants 29 to < 35 weeks gestation against RSV LRTI. However, most medically attended (MA) cases occur in otherwise healthy, term infants for whom there is currently no effective RSV prevention strategy. We report the primary analysis of efficacy and safety, along with the impact of nirsevimab in late preterm and term infants (≥ 35 weeks gestation) in the phase 3 MELODY study (NCT03979313).

**Methods:**

Infants were randomized 2:1 to receive one intramuscular injection of nirsevimab (50 mg if < 5 kg; 100 mg if ≥ 5 kg at dosing) or placebo entering their first RSV season. The primary endpoint was the incidence of MA RSV LRTI over 150 days postdose. Cases met predefined clinical criteria of disease severity and were confirmed by real-time reverse-transcriptase PCR. Safety was evaluated through 360 days postdose. Enrollment started on 23 July 2019 and was suspended following the declaration of the COVID-19 pandemic by the WHO on 11 March 2020.

**Results:**

Overall, 1490 infants were randomized and included in the intent-to-treat population; 1465 (98%) completed the 150-day efficacy follow-up, and 1367 (92%) completed the 360-day safety follow-up. The incidence of MA RSV LRTI was 1.2% (n=12/994) in the nirsevimab group and 5.0% (n=25/496) in the placebo group, giving nirsevimab an efficacy of 74.5% (95% confidence interval [CI]: 49.6, 87.1; p< 0.0001). Nirsevimab averted 93.6 (95% CI 63.0, 124.0) MA LRTIs per 1000 infants dosed. Nirsevimab was well tolerated, with similar rates of adverse events (87.4% nirsevimab; 86.8% placebo) and serious adverse events (6.8% nirsevimab; 7.3% placebo) between groups.

**Conclusion:**

In this phase 3 study, a single dose of nirsevimab protected late preterm and term infants against MA RSV LRTI over an RSV season with a favorable safety profile. Approximately 11 infants need to be immunized to prevent 1 case of LRTI; nirsevimab has the potential to be an important intervention to reduce the burden of RSV LRTI in healthy infants.

**Disclosures:**

**Laura Hammitt, MD**, **MedImmune** (Grant/Research Support, Scientific Research Study Investigator, Research Grant or Support)**Merck & Co., Inc.** (Grant/Research Support, Scientific Research Study Investigator, Research Grant or Support)**Novavax** (Grant/Research Support, Scientific Research Study Investigator, Research Grant or Support)**Pfizer** (Grant/Research Support, Scientific Research Study Investigator, Research Grant or Support) **Laura Hammitt, MD**, MedImmune (Individual(s) Involved: Self): Grant/Research Support, Research grant to my institution; Merck (Individual(s) Involved: Self): Grant/Research Support, Research grant to my institution; Pfizer (Individual(s) Involved: Self): Grant/Research Support, Research grant to my institution **Ron Dagan, MD**, **Medimmune/AstraZeneca** (Grant/Research Support, Scientific Research Study Investigator, Research Grant or Support)**MSD** (Consultant, Grant/Research Support, Scientific Research Study Investigator, Advisor or Review Panel member, Research Grant or Support, Speaker’s Bureau)**Pfizer** (Consultant, Grant/Research Support, Scientific Research Study Investigator, Advisor or Review Panel member, Research Grant or Support, Speaker’s Bureau) **Yuan Yuan, PhD**, **AstraZeneca** (Employee, Shareholder) **Shabhir A. Mahdi, PhD**, **BMGF** (Research Grant or Support)**EDCTP** (Research Grant or Support)**GlaxoSmithKline** (Research Grant or Support)**Melody** (Research Grant or Support)**Minervax** (Research Grant or Support)**Novavax** (Research Grant or Support)**SAMRC** (Research Grant or Support) **William J. Muller, MD, PhD**, **Ansun** (Scientific Research Study Investigator)**Astellas** (Scientific Research Study Investigator)**AstraZeneca** (Scientific Research Study Investigator)**Genentech** (Scientific Research Study Investigator)**Gilead** (Scientific Research Study Investigator)**Janssen** (Scientific Research Study Investigator)**Karius** (Scientific Research Study Investigator)**Melinta** (Scientific Research Study Investigator)**Merck** (Scientific Research Study Investigator)**Nabriva** (Scientific Research Study Investigator)**Seqirus** (Scientific Research Study Investigator)**Tetraphase** (Scientific Research Study Investigator) **William J. Muller, MD, PhD**, Ansun (Individual(s) Involved: Self): Grant/Research Support; Astellas (Individual(s) Involved: Self): Research Grant or Support; AstraZeneca (Individual(s) Involved: Self): Grant/Research Support; BD (Individual(s) Involved: Self): Research Grant or Support; Eli Lilly (Individual(s) Involved: Self): Grant/Research Support; Gilead (Individual(s) Involved: Self): Grant/Research Support; Karius, Inc. (Individual(s) Involved: Self): Grant/Research Support, Scientific Research Study Investigator; Melinta (Individual(s) Involved: Self): Grant/Research Support; Merck (Individual(s) Involved: Self): Grant/Research Support; Moderna (Individual(s) Involved: Self): Grant/Research Support; Nabriva (Individual(s) Involved: Self): Grant/Research Support; Seqirus (Individual(s) Involved: Self): Consultant; Tetraphase (Individual(s) Involved: Self): Grant/Research Support **Heather J. Zar, PhD**, **AstraZeneca** (Grant/Research Support)**Novavax** (Grant/Research Support)**Pfizer** (Grant/Research Support, Advisor or Review Panel member) **Dennis Brooks, MD**, **AstraZeneca** (Employee) **Amy Grenham, MSc**, **AstraZeneca** (Employee, Shareholder) **Ulrika Wählby Hamrén, PhD**, **AstraZeneca R&D** (Employee, Shareholder) **Vaishali S. Mankad, MD**, **AstraZeneca** (Employee) **Therese Takas, BSc**, **AstraZeneca** (Employee, Other Financial or Material Support, Own stock in AstraZeneca) **Jon Heinrichs, PhD**, **AstraZeneca** (Shareholder)**Bristol Myers Squibb** (Shareholder)**J&J** (Shareholder)**Merck** (Shareholder)**Organon** (Shareholder)**Procter & Gamble** (Shareholder)**Sanofi** (Shareholder)**Sanofi Pasteur** (Employee) **Amanda Leach, MRCPCH**, **AstraZeneca** (Employee, Shareholder) **M. Pamela Griffin, MD**, **AstraZeneca** (Employee) **Tonya L. Villafana, PhD**, **AstraZeneca** (Employee)

